# And Then There Were Three…: Extreme Regeneration Ability of the Solitary Chordate *Polycarpa mytiligera*

**DOI:** 10.3389/fcell.2021.652466

**Published:** 2021-04-15

**Authors:** Tal Gordon, Arnav Kumar Upadhyay, Lucia Manni, Dorothée Huchon, Noa Shenkar

**Affiliations:** ^1^School of Zoology, George S. Wise Faculty of Life Sciences, Tel-Aviv University, Tel-Aviv, Israel; ^2^Department of Biology, University of Padua, Padua, Italy; ^3^The Steinhardt Museum of Natural History, Israel National Center for Biodiversity Studies, Tel-Aviv University, Tel-Aviv, Israel

**Keywords:** tunicates, regenerative biology, cell proliferation, phylogeny, *Polycarpa*, evolution

## Abstract

Extensive regenerative ability is a common trait of animals capable of asexual development. The current study reveals the extraordinary regeneration abilities of the solitary ascidian *Polycarpa mytiligera*. Dissection of a single individual into separate fragments along two body axes resulted in the complete regeneration of each fragment into an independent, functional individual. The ability of a solitary ascidian, incapable of asexual development, to achieve bidirectional regeneration and fully regenerate all body structures and organs is described here for the first time. Amputation initiated cell proliferation in proximity to the amputation line. Phylogenetic analysis demonstrated the close affinity of *P. mytiligera* to colonial species. This evolutionary proximity suggests the ability for regeneration as an exaptation feature for colonial lifestyle. *P. mytiligera*’s exceptional regenerative abilities and phylogenetic position highlight its potential to serve as a new comparative system for studies seeking to uncover the evolution of regeneration and coloniality among the chordates.

## Introduction

Regeneration is widespread across metazoans, with representatives from most phyla presenting various degrees of ability to reconstruct lost or damaged body parts. However, the evolution of regeneration and the factors that determine its distribution across the animal kingdom are far from being clear (Sánchez Alvarado, 2000; Giangrande and Licciano, 2014).

Most animals, including humans, regenerate specific cell types, such as the epidermis and hair cells, on a daily basis. The ability to regenerate large and complex body structures, however, differs among the metazoans. Most, if not all, cnidarians and poriferans present high regenerative abilities, while other groups, such as the nematodes, have lost the ability to regenerate almost all cell types. Most striking are the groups in which closely related species present very different regenerative properties, as observed in the annelids (Galliot and Schmid, 2002; Alvarado and Tsonis, 2006; Bely, 2006; Tanaka and Reddien, 2011). Members of the latter group have been studied extensively and provide a unique view of the mechanisms behind the loss or gain of regeneration abilities (Bely, 2006).

The phylogenetic distribution of regeneration across the metazoans raises the possibility of this ability being an ancestral feature, and emphasizes the importance of comparative studies employing new model systems in order to gain insight into the evolution of regeneration and the mechanisms that determine its variation across species (Bely and Nyberg, 2010; Giangrande and Licciano, 2014; Grillo et al., 2016; Slack, 2017; Ricci and Srivastava, 2018; Mehta and Singh, 2019).

Ascidians are sessile invertebrates found in all marine habitats, from shallow waters to the deep sea (Monniot et al., 1991; Shenkar and Swalla, 2011). Their phylogenetic position as a sister group of the vertebrates, simple body plan, and exceptional regenerative abilities (Voskoboynik et al., 2007; Brown et al., 2009; Kassmer et al., 2020), have established them as a valuable model system for developmental and evolutionary studies (Satoh and Jeffery, 1995; Corbo et al., 2001; Passamaneck and Di Gregorio, 2005; Delsuc et al., 2006; Voskoboynik and Weissman, 2015; Kowarsky et al., 2019; Manni et al., 2019).

The majority of solitary ascidians reproduce sexually by releasing gametes into the seawater for external fertilization, while colonial species also propagate by asexual reproduction or budding (Berrill, 1975). These asexual strategies has evolved independently several times across the Ascidiacea, and has been shown to involve a variety of cellular mechanisms in different species (Gutierrez and Brown, 2017; Manni et al., 2019; Scelzo et al., 2019; Ferrario et al., 2020). Furthermore, some colonial species exhibit more than one budding strategy, activated by different stimuli (Manni et al., 2019). In addition to these developmental processes, which are part of their life cycle, both solitary and colonial ascidians share another mode of development in the form of tissue and whole-body regeneration (Gordon et al., 2019; Kassmer et al., 2019; Alié et al., 2020). Colonial species possess high regenerative abilities, being able to regenerate their entire body from small tissue fragments or blood cells (Voskoboynik et al., 2007; Brown et al., 2009; Blanchoud et al., 2017; Kassmer et al., 2020). In contrast, the few solitary species studied to date have demonstrated a more limited ability, as most can only regenerate anterior organs following their removal, such as the siphons and neural complex (Jeffery, 2015; Medina et al., 2015; Gordon et al., 2019).

A high correlation between asexual reproduction and robust regeneration abilities following injury have also been found in other animal groups such as the annelids and cnidarians (Bely, 2006; Giangrande and Licciano, 2014; Sánchez Alvarado and Yamanaka, 2014). While this correlation suggests that both processes share common basic genetic pathways (Brockes et al., 2001; Grillo et al., 2016), it raises questions regarding the evolution of regeneration properties in solitary organisms, incapable of asexual development. These organisms, while relying on sexual reproduction, also possess the cellular and genetic tool kit needed for tissue and organ regeneration. The diversity of developmental pathways and regenerative properties found in phylogenetically related species makes the ascidians a highly interesting group for evolutionary and regenerative studies (Brown and Swalla, 2012; Jeffery, 2016; Alié et al., 2018). However, resolving the phylogenetic relationships among these species is first required, in order to accurately assess the evolutionary dynamics that determines the regeneration capabilities in the Ascidiacea (Bely and Nyberg, 2010).

Recent studies have demonstrated that regeneration in both colonial and solitary ascidians involves the proliferation of pluripotent or multipotent cells that eventually give rise to a blastema (Jeffery, 2014, 2019; Kassmer et al., 2016). Kassmer et al. (2020) showed the presence of blood-borne multipotent cells in the colonial *Botrylloides leachii* (order Stolidobranchia) and demonstrated their functional role in whole-body regeneration. Similarly, in the solitary ascidian *Ciona intestinalis* (order Phlebobranchia), branchial sac piwi-positive hemocytes were shown to migrate and proliferate in response to injury (Jeffery, 2014). Despite these similarities in the cellular and molecular processes underling regeneration in colonial and solitary ascidians, members of these two groups display major differences in regeneration ability.

The current study focuses on the solitary ascidian *Polycarpa mytiligera* (order: Stolidobranchia). Our recent studies have demonstrated this species’ unusual ability to regenerate both distal structures and internal organs (Shenkar and Gordon, 2015; Gordon and Shenkar, 2018; Gordon et al., 2019). In the present work, we present this species extraordinary ability of regenerating all its body structures and tissue types into separate, functional individuals following amputation along two major body axes. The ability to regenerate a whole animal from small body fragments distinguishes *P. mytiligera* from other solitary species. Transcriptome-scale phylogenetic analysis indicated close relationship between this highly regenerative solitary species and asexual, colonial ascidians. Our results present *P. mytiligera* as a new and promising model for regeneration and developmental research, and highlight the importance of this organism in the study of the evolution of chordate regeneration.

## Materials and Methods

### Animal Collection and Culturing

*P. mytiligera* adults were collected from the Gulf of Aqaba (Eilat), Red Sea, Israel. The animals were maintained at the Inter-University Institute (IUI) in aquaria with running seawater for 4 days of acclimation prior to onset of the experiments. All individuals were of a similar size-range (2.5 ± 1 cm, from the tip of the oral siphon to the base of the animal).

*P. mytiligera* juveniles (3 months old, 5 ± 1 mm) were obtained from a breeding culture established at the IUI (Gordon et al., 2020).

### Surgical Procedures

**FIGURE 1:**
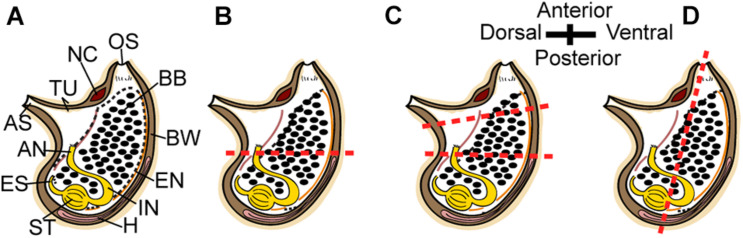
Regeneration experiment. **(A–D)** Scheme of *P. mytiligera* adult (right view). **(B)** amputation at a single level along the AP body axis. **(C)** Amputation at two levels along the AP body axis. **(D)** amputation at a single level along the DV body axis. The red dotted line indicates amputation level. Anus (AN), atrial siphon (AS), branchial basket (BB), body wall (BW), endostyle (EN), esophagus (ES), heart (H), intestine (IN), neural complex (NC), oral siphon (OS), stomach (ST), and tunic (TU).

To determine *P. mytiligera*’s regenerative ability adult animals were amputated at different levels along two body axes: (a) amputation at a single level along the anterior posterior (AP) body axis; (b) amputation at two levels along the AP body axis; and (c) amputation at a single level along the dorsal ventral (DV) body axis (Figure 1). Following anesthetization with menthol crystals in seawater (Stefaniak and Heupel, 2016), animals (*n* = 10 per experiment) were amputated using a scalpel (Bar Naor, #BN400-11-JH). All body parts were then labeled according to the relevant experiment. Regenerating and non-amputated control animals (*n* = 10) were maintained in an open water system with running seawater (Gordon et al., 2020) and photographed weekly for 40 days. Feeding was provided by the natural food particles entering the system via the water current. Response to touch was used to determine survival.

### Histology

In order to study the effect of amputation level on regeneration efficiency and timing, adult animals were amputated as described in Figure 1. Body fragments were fixed at different time points along the regeneration process: amputation day, 7, 30, and 40 days post-amputation (dpa) (*n* = 3 per time point). Animals were fixed in 4% formalin in seawater after relaxation in menthol, separated partly from the tunic, and dehydrated in ethanol at increasing concentrations prior to paraffin embedding (Paraplast Plus, Leica). Sections (7 μm) were mounted on glass slides, deparaffinized, and stained with standard hematoxylin and eosin solutions.

### *In vivo* Cell Labeling Experiments

Cell proliferation was detected by the incorporation of 5-ethynyl-2-deoxyuridine (EdU) into replicating DNA. *P. mytiligera* juveniles (3 months old) were divided into four groups and bisected midway along the AP body axis (Figure 1B). Each group was exposed to a EdU pulse at a different time point (pre-amputation, amputation day, 5 and 10 days post-amputation) along the regeneration process (*n* = 3 per each time point).

For the pulse experiments animals were incubated with 10 μmol/L EdU (Invitrogen, Carlsbad, CA) in 5 mL of MFSW for 16 h in Petri dishes. Following completion of the labeling, animals were fixed for 12 h in 4% PFA, rinsed three times in 1 × phosphate-buffered saline (PBS), and processed for EdU detection using Alexa Fluor azide 488 at room temperature, according to the instructions of the Click-iT EdU Alexa Fluor High Throughput Imaging Assay Kit (Invitrogen). Samples were stained with DAPI (Thermo Fisher Scientific 33342) (1 μg/mL in PBS), and mounted in VECTASHIELD (Vector Laboratories RK-93952-28) using coverslips.

### Image Acquisition and Processing

Images of whole mount specimens were taken using a Zeiss LSM 880 scanning laser confocal microscope. Maximum intensity Z-projections and cell counting were generated using the Fiji image processing software.

Quantification of EdU labeled cells was done by counting the cells in 100 μm^2^ z-stack regions of control and regenerating animals. Labeled cells were counted in regions proximal and distal to the amputation line (proximality was determined as <500 μm from the amputation line). Labeled cells were also counted in two body structures: the branchial basket and the body wall. Counts were averaged for each sample (*n* = 3 biological, 3 technical replicates). The percentage of EdU-positive cells was calculated from the total number of DAPI positive cell nuclei counted per each region.

### Transcriptome Sequencing and Phylogenetic Analysis

*De novo* transcriptome sequencing was performed on a single *P. mytiligera* adult. Total RNA was extracted from the whole body (excluding the tunic and digestive system) using the RNAeasy mini kit, (Qiagen, Germany). The cDNA libraries preparation and paired-ended sequencing were performed by The Technion Genome Center (Haifa, Israel), using an Illumina HiSeq 2000 machine. The 100 base pairs reads were filtered from adapters using the CutAdapt version 1.16 program (Martin, 2011) and quality checks were performed with the program FastQC version 0.11.5 (Andrews, 2010). Transcriptome assembly of the filtered reads was performed with Trinity (v2.8.4) under the parameters: –min_contig_length 150 –min_kmer_cov 5 (Grabherr et al., 2011). The program TransDecoder (Release v5.5.0) (Haas et al., 2013) was used to identify and translate the longest possible transcript from each *P. mytiligera* contig.

To reconstruct the phylogenetic position of *P. mytiligera* we used the dataset of Alié et al. (2018), which comprises 4,908 protein-coding gene alignments in tunicates (available at https://github.com/AlexAlie/styelidae). We extracted from each alignment one representative sequence: *Polycarpa pomaria* (4,519 alignments), *Polycarpa mamillaris* (140 alignments), *Polyandrocarpa zorritensis* (125 alignments), *Polycarpa* sp. (63 alignments), *Halocynthia roretzi* (35 alignments), *Polycarpa aurata* (17 alignments), *Styela plicata* (8 alignments), and *Distomus variolosus* (1 alignment). These sequences were used as query in a blastp search against the translated transcriptome assembly of *P. mytiligera*, using a cutoff –e value of 1e-5 and the options -max_hsps 1 and -max_target_seqs 1. The *P. mytiligera* hits obtained were used as query in a reciprocal tblastn search against the transcriptome of the corresponding species using the same cutoff and options as above. Each transcriptome was downloaded from https://github.com/AlexAlie/styelidae. In 789 cases the reciprocal search did not lead to the sequence at the origin of the first search. The corresponding alignments were thus excluded from the analyses. Despite the reciprocal filtering, some sequences were assigned to more than one alignment file. Again, the corresponding 746 alignments were removed. Finally, we searched for putative contaminations, using the selected *P. mytiligera* transcripts selected. To do so, the *P. mytiligera* transcripts were used as queries in a blastn and blastx search against the NCBI nr database (last accessed 10 July 2019). No high similar hits to bacterial, *Homo sapiens*, or other eukaryotic sequences were detected. Following completion of the filtering steps, 3,373 alignments were retained.

For each alignment, ambiguously aligned positions were removed using a standalone version of GUIDANCE (v2.02) (Sela et al., 2015). GUIDANCE was run under default settings using MAFFT as the alignment program option. A total of 32 alignments were excluded during the GUIDANCE step. The filtered alignments were then concatenated with a python code. Two data matrix were considered, the first comprised all 3,341 protein genes; and the second, also termed “reduced dataset,” comprised 182 genes that did not have missing taxa. Phylogenetic reconstructions were performed on these two concatenated alignments (i.e., the all genes dataset and the reduced dataset) with the program IQ-Tree v. 1.6.12 (Nguyen et al., 2015). First, a guide tree was obtained using the LG + F + G model. Using the tree obtained under this simple model we then used the posterior mean site frequency (PMSF) LG + C60 + F + G model to reconstruct relationships. This model was chosen since it is among the most robust models against branch-attraction artifacts, and also saves computation time compared to Bayesian alternatives (Wang et al., 2018). Branch supports were computed using 100 standard non-parametric bootstrap replicates.

### Statistics

Boxplots prepared with R (R 3.6.1) using ggplot2 packages (v. 3.2.1; Wickham, 2016). Statistical tests were performed in R (see Figure legends for details).

## Results

### *Polycarpa mytiligera* is Capable of Regenerating Its Entire Body From a Small Fragment

**FIGURE 2:**
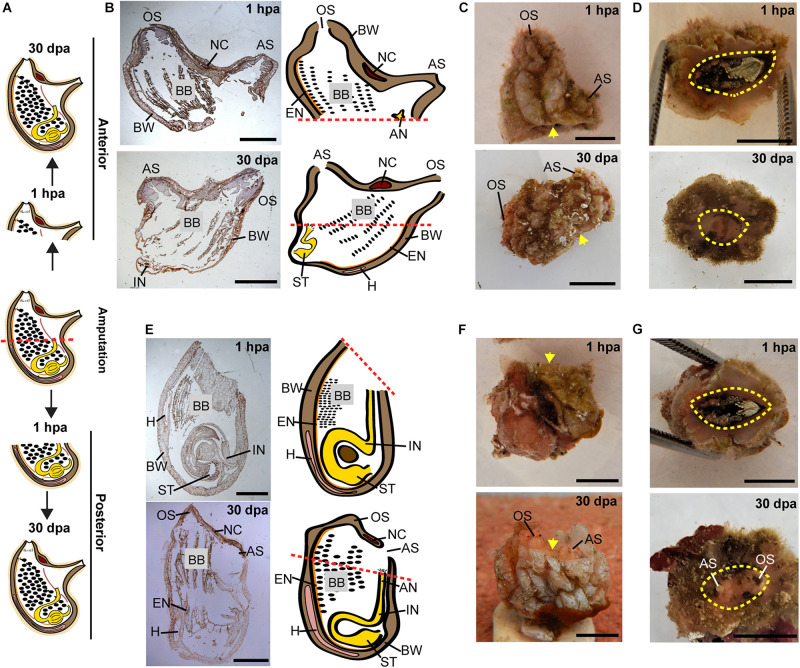
Regeneration along the AP axis into two separate animals is complete by 30 dpa. **(A)** Illustration depicting the regeneration process at 1-h post amputation (hpa) and 30 dpa. **(B–D)** Regeneration of the posterior body part from the anterior fragment. **(B)** Section left view and matching illustration. Red dashed lines indicate amputation line. **(C,D)** *In vivo* images. **(C)** Left view. Yellow arrow indicates wound area. **(D)** Bottom view. Yellow dashed lines indicate wound border. **(E–G)** Regeneration of the anterior body part from the posterior fragment. **(E)** Section left view and matching illustration. **(F,G)** *In vivo* images. **(F)** Left view. **(G)** Top view. Anus (AN), atrial siphon (AS), body wall (BW), branchial basket (BB), endostyle (EN), heart (H), intestine (IN), neural complex (NC), oral siphon (OS), stomach (ST). Scale bar in **(C,D,F,G)**: 0.5 cm, and in: **(B,E)**: 3 mm (see also Supplementary Table 1 and Supplementary Figure 1).

**FIGURE 3:**
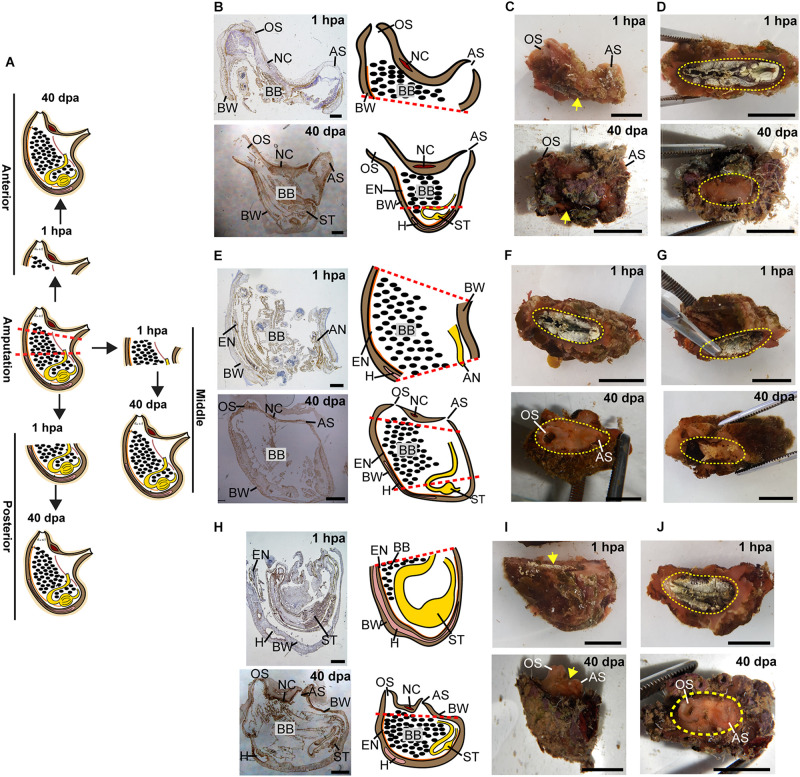
Regeneration along the AP axis into three separate animals is complete by 40 dpa. **(A)** Illustration depicting regeneration process at 1 hpa and 40 dpa. **(B–D)** Regeneration of the posterior body part from the anterior fragment. **(B)** Sections left view and matching drawing. Red dashed lines indicate amputation line. **(C)** *In vivo* images, left view. Yellow arrow indicates wound area. **(D)** *In vivo* images, bottom view. Yellow dashed lines indicate wound border. **(E–G)** Regeneration of the anterior and posterior body parts from the middle body part. **(E)** Sections left view and matching drawing. **(F,G)** *In vivo* images of both sides of the animal. **(F)** Top view. **(G)** Bottom view. **(H–J)** Regeneration of the anterior body part from the posterior fragment. **(H)** Sections left view and matching drawing. **(I)** *In vivo* images, left view. **(J)** *In vivo* images, Top view. Anus (AN), atrial siphon (AS), body wall (BW), branchial basket (BB), endostyle (EN), heart (H), intestine (IN), neural complex (NC), oral siphon (OS), stomach (ST). Scale bar in **(C,D,F,G,I,J)**: 1 cm, and in: **(B,E,H)**: 2 mm (see also Supplementary Table 2 and Supplementary Figure 2).

**FIGURE 4:**
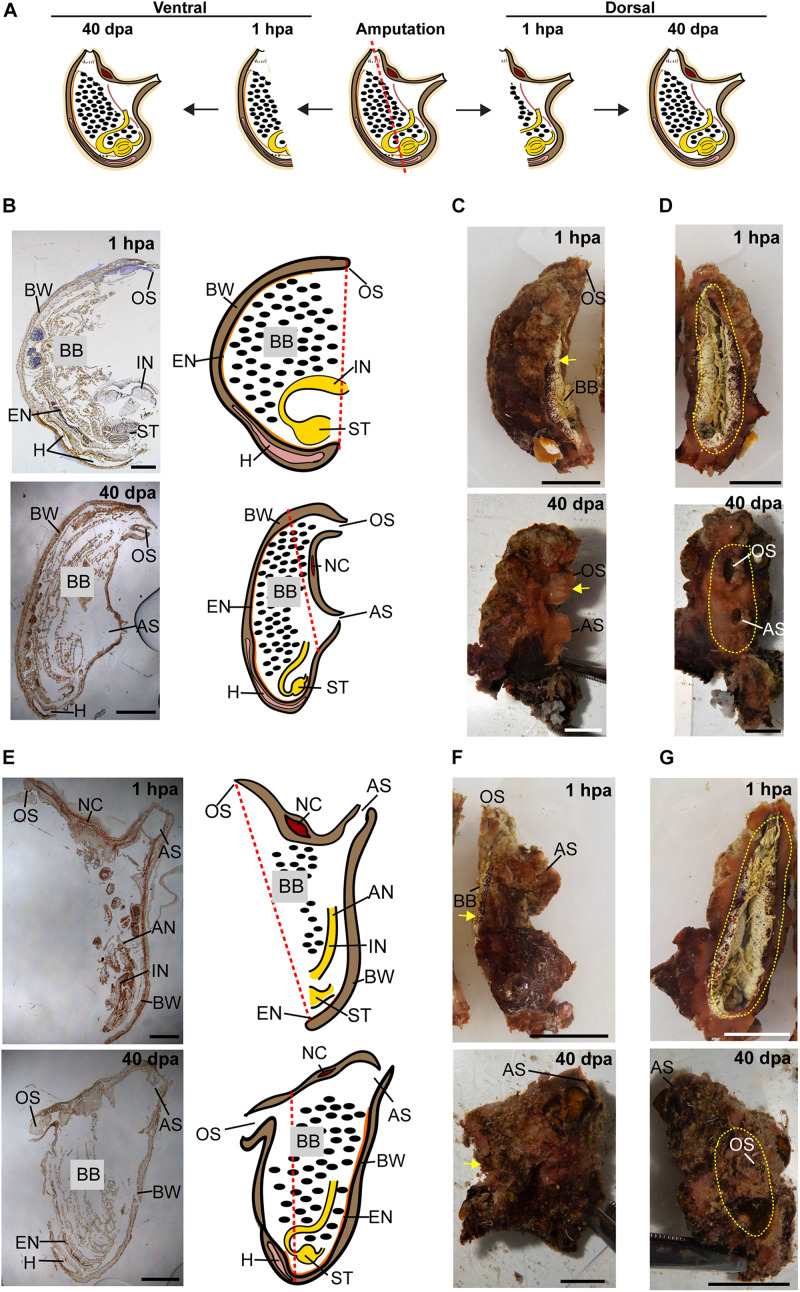
Regeneration along the DV axis of both body fragments is complete by 40 dpa. **(A)** Illustration depicting regeneration along the DV axis at 1 hpa and 40 dpa. **(B–D**) Ventral body part. **(B)** Sections left view and matching drawing. Red dashed lines indicate amputation line. **(C)** *In vivo* images, left view. Yellow arrow indicates wound area. **(D)** *In vivo* images, posterior view. Yellow dashed lines indicate wound border. **(E–G)** Dorsal body part. **(E)** Sections left view and matching drawing. **(F)** *In vivo* images, left view. **(G)** *In vivo* images, posterior view. Anus (AN), atrial siphon (AS), body wall (BW), branchial basket (BB), endostyle (EN), heart (H), intestine (IN), neural complex (NC), oral siphon (OS), stomach (ST). Scale bar in **(C,D,F,G)**: 0.5 cm, and in: **(B,E)**: 3 mm (see also Supplementary Table 3 and Supplementary Figure 3).

All the tested animals survived the amputation along two body axes and initiated regeneration in all body fragments (Figure 1). Amputation resulted in a wound-healing process, evidenced by the formation of epidermal tissue in the regenerating area, recognizable both in the live animals and in the histological sections (Figures 2–4 and Supplementary Figures 1–3). By 30–40 days post-amputation (dpa), all fragments had fully regenerated into separate individuals (Figures 2–4 and Supplementary Figures 1–3). While the overall morphology of the regenerated animals was generally similar to that of the non-amputated control animals, the regenerated animals were smaller in size. Depending on the amputation level, the regenerating fragments were about a half or a third of the size of the original animal. The dissected animals were able to regenerate all their tissue types and organs following their removal (Supplementary Tables 1–3). Analysis of serial sections of entire animals at the different time points of the regeneration process enable detailed characterization of the organs that remains in each body part following amputation, and the new organs that re-formed during the regeneration process (see Supplementary Tables 1–3 for summary). While some structures, as the body wall and the branchial basket re-grew from the residual tissues, other body systems such as the neural complex, digestive system, and heart regenerated as newly formed parts that retained no residues of the original organs (Figures 2–4, Supplementary Figures 1–3, and Supplementary Tables 1–3). At the end of the experiments, all the newly formed animals responded to external stimuli by body or siphon contractions.

### Cell Proliferation Increases During Anterior Regeneration

**FIGURE 5:**
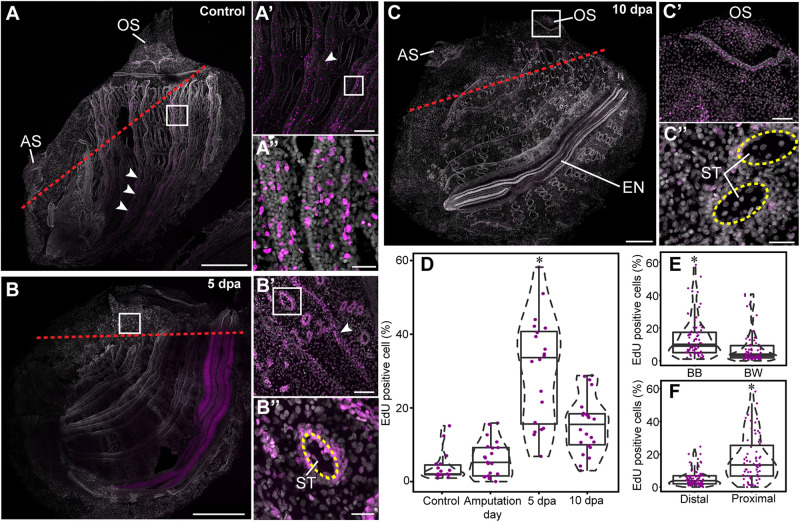
Cell proliferation increase during anterior regeneration. Whole mount EdU staining images of anterior regeneration. EdU labeling in magenta and DAPI nuclear staining in gray. All panels are anterior to the top, right view. **(A,A′,A″)** Unamputated control. **(A′)** Branchial basket, enlargement of square area in **(A)**. **(A″)** Longitudinal vessels, enlargement of square area in **(A′)**. Red dashed lines outline the amputation line. White arrowheads indicate longitudinal vessels. **(B,B′,B″)** 5 dpa. **(B′)** Branchial basket, enlargement of square area in **(B)**. **(B″)** Stigma, enlargement of the square area in **(B′)**. Yellow dashed lines outline the stigma. **(C,C′,C″)** 10 dpa. **(C′)** Oral siphon, enlargement of square area in **(C)**. **(C″)** Stigmata. **(D–F)** Quantification of EdU-positive cells in 100 μm^2^ sections. Violin plots display the proportion (%) of EdU-positive cells in relation to total number of DAPI positive cell nuclei. The plot diameter reflects the probability density of EdU-positive cells for each time point. Jittered points overlaid on boxplots represent individual samples. The bottom and top of the boxes represent the first and third quartiles, the central band represents the median, and whiskers represent 1.5-fold the interquartile range. *P*-values calculated with the Mann Whitney Wilcoxon test are indicated above each boxplot. **(D)** Percentage of EdU-positive cells proximal to the amputation line along the different time points (*n* = 3 per time point). **(E)** Percentage of EdU-positive cells (*n* = 15) in the branchial basket (BB) and body wall (BW). **(F)** Percentage of EdU-positive cells (*n* = 9) in proximal and distal areas from the amputation line. Atrial siphon (AS), endostyle (EN), oral siphon (OS), and stigmata (ST). Scale bar: **(A,B)** 500 μm; 200; **(A’,C)** 100 μm; **(B′,C′)** 50 μm; **(A″,B″,C″)** 20 μm (see also Supplementary Figure 4).

Juveniles were bisected midway along the anterior-posterior (AP) axis (Figure 1B). Both resulting fragments were completely regenerative, restoring all lost tissues and organs over the course of 2 weeks. Although regenerative response was apparent in both fragments, particular attention was devoted to the regeneration of the posterior fragments, and the experimental design was aimed at visualizing the location of proliferating cells at the desired stages of the regeneration process. EdU incorporation was determined at three stages: (a) early regeneration, 16 h following amputation; (b) mid regeneration, 5 dpa, following closure of the wound by epidermal cells and prior to anterior organ differentiation; and (c) late regeneration, 10 dpa, when the anterior organs were fully regenerated (Figure 5 and Supplementary Figure 4).

To determine whether cell proliferation post amputation was restricted to the regenerating area or, rather, a wider process occurred simultaneously across the animal’s entire body, we analyzed cell proliferation in regions proximal and distal to the amputation line (Figure 5 and Supplementary Figure 4). A proximal region was determined as <500 μm from the amputation line. In addition, based on previous studies that had identified the branchial basket circulatory cells (hemocytes) as involved in distal regeneration in the solitary ascidian *C. intestinalis* (Jeffery, 2019), we examined cell proliferation levels in this structure in comparison with the body wall at different stages of regeneration (Figure 5).

Following amputation, proliferating cells were unevenly distributed in *P. mytiligera*’s body. A higher level of EdU+ cells was found in the branchial basket along all stages of the regeneration process in comparison to that of the body wall (Figure 5E). Furthermore, a higher level of EdU positive cells was found in proximity to the amputation line (Figure 5F). In distal areas, the level of dividing cells remained low, showing no significant difference between the different time points.

In the control animals, EdU incorporation was detected in the siphons, branchial basket, endostyle, and digestive system (Figures 5A–A″). No difference was found in the level of EdU+ cells among the different tissue types. On amputation day, the level of EdU+ cells was similar to that of the control in both proximal and distal regions, suggesting no major impact of amputation on cell proliferation at this early stage. By 5 dpa (Figures 5B–B″), EdU+ cell levels was significantly higher in areas proximal to the amputation line in tissue layers within both the body wall and branchial basket (Figure 5D), indicating an accumulation of dividing cells close to the regeneration area. At 10 dpa (Figures 5C–C″) EdU+ cell levels had decreased although remaining higher than those of the control (Figure 5D).

The endostyle showed specific labeling at different regeneration stages, with a strong signal at 5 dpa (Figure 5 and Supplementary Figure 4). However, the organ’s complex morphology limited our ability to accurately distinguish labeled cells for quantitative analysis.

Overall, these results indicate that anterior amputation triggers cell division in a proliferation zone proximal to the amputation plane, and raises the possibility that this process is a necessary step in the course of successful regeneration.

### *P. mytiligera* Demonstrates a Close Phylogenetic Relationship to Colonial Species

**FIGURE 6:**
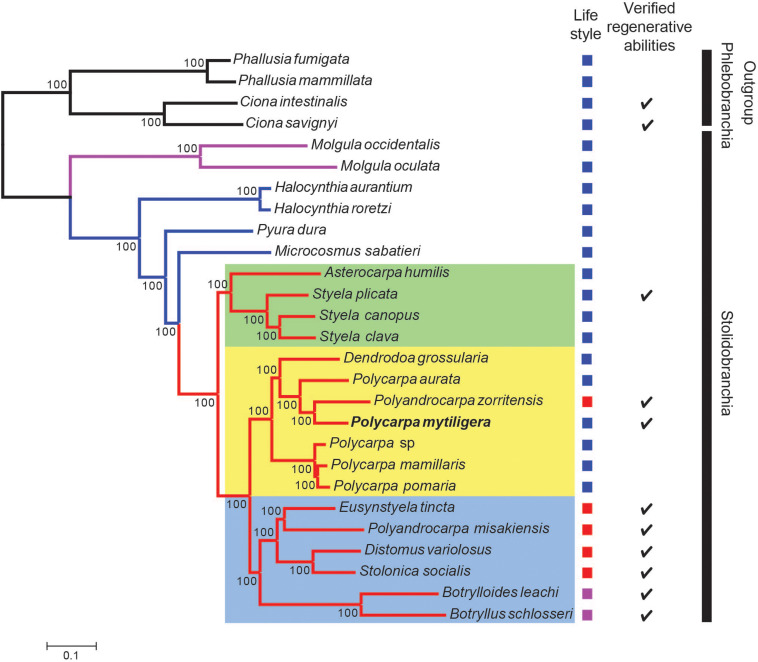
*P. mytiligera* demonstrates a close phylogenetic relationship to colonial species. Phylogenetic relationships between Stolidobranchia, with a focus on Styelidae, inferred from 182 protein coding genes under the LG + C60 + F + G model. Newly sequenced *P. mytiligera* is shown in bold. Bootstrap supports are indicated near the corresponding nodes. Red branches indicate Styelidae species, blue Pyuridae species, purple Molgulidae species, and black Phlebobranchia species used as outgroup. Colored squares represent different clades. Left column: life-style modes as documented in the literature (blue: solitary, purple: colonial, red: colonial with separated zooids). Right column: checkmark indicates species where regenerative ability were documented in the literature (see also Supplementary Figure 5).

The two phylogenetic trees reconstructed with either 3,341 genes (Supplementary Figure 5) or the reduced dataset (Figure 6) were identical and agree with Alié et al. (2018). In these two trees, all branches present maximal bootstrap support values (bootstrap percentage, *BP* = 100). Specifically, the Molgulidae are the first Stolidobranchia family to diverge and the Pyuridae are paraphyletic (Figure 6). Within the Styelidae, two distinct clades were found: one strictly solitary (green square, Figure 6), containing *Styela* and *Asterocarpa*, and the other a mixture of colonial and solitary species. In this second clade, colonial and solitary species are divided into two clades. The first clade (blue square, Figure 6) contains only colonial species and comprises the Botryllinae (*Botryllus schlosseri* and *Botrylloides leachii*), as a sister group of members of the Polyzoinae (*Polyandrocarpa misakiensis*, *Eusynstyela tincta*, *Distomus variolosus*, and *Stolonica socialis*). The second clade (yellow square, Figure 6) comprises a paraphyletic *Polycarpa*, which encompasses *Dendrodoa grossularia* and the colonial Polyzoinae *Polyandrocarpa zorritensis*. Within the *Polycarpa* clade, *Polycarpa mytiligera* is sister to *Polyandrocarpa zorritensis.*

## Discussion

Ascidians are unique among the chordates as they present robust regeneration abilities, including whole body regeneration, following an injury (Voskoboynik et al., 2007; Blanchoud et al., 2018; Kassmer et al., 2020). The current study has uncovered exceptional regeneration abilities in a solitary ascidian, a group of animals considered to be regeneratively deficient compared to their colonial relatives (Kassmer et al., 2019). *P. mytiligera*’s provides a valuable opportunity to elucidate the evolution of coloniality and reveal conserved pathways that regulate chordate regeneration.

### *P. mytiligera* Regeneration Involves Extensive Cell Proliferation and Reorganization Processes

*P. mytiligera* presented an extraordinary regeneration plasticity, being able to reconstruct an entire animal from each small body fragment. Histological sections confirmed that despite no residue of essential organs, such as the heart and neural complex, remaining in the body fragment, the animal was nonetheless able to regenerate and regain its complete morphology and functionality.

**FIGURE 7:**
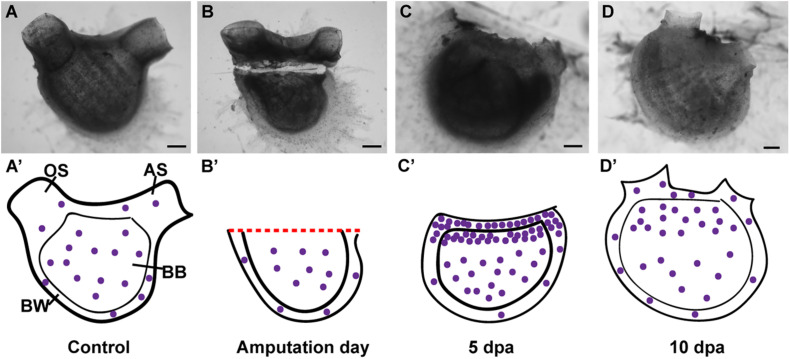
Summary of proliferating cell dynamic in *P. mytiligera* inferred from EdU experiments. **(A,A′)** Control. **(A)** *In vivo* image, **(A′)** illustration. During homeostasis, proliferating cells (purple spheres) are evenly dispersal along the branchial basket (BB) and body wall (BW) [Oral siphon (OS), atrial siphon (AS)]. **(B,B′)** Amputation day. **(B)** *In vivo* image, **(B′)** illustration. The level of proliferating cells remains similar to that of the control. **(C,C′)** 5 days following anterior amputation. **(C)** *In vivo* image, **(C′)** illustration. Proliferating cells specifically accumulate at the amputation site in the branchial basket and body wall in proximity to the regenerating area. **(D,D′)** 10 days following amputation. **(D)** *In vivo* image, **(D′)** illustration. As the amputated structures regenerated, the level of proliferating cell decreased in relation to 5 dpa, although remaining higher than those of the control. Scale bar in **(A,B)**: 1 mm, and in: **(C,D)**: 500 μm.

*P. mytiligera*’s regeneration process can be divided into three phases: (1) wound-healing; (2) increased cell proliferation in the injured area; and (3) morphogenesis and formation of tissues and organs. These basic stages, underlying tissue regeneration, are highly conserved, being found in a wide variety of animals capable of regeneration (Alvarado and Tsonis, 2006; Ricci and Srivastava, 2018). In planarians, regeneration involves the assembly of a blastema composed of pluripotent somatic stem cells. Limb regeneration in salamanders and fin regeneration in zebrafish also require the formation of a blastema; however, there the blastema is composed of a mixture of cells with different and restricted potentials (Alvarado and Tsonis, 2006; Sánchez Alvarado, 2006; Tanaka and Reddien, 2011; Tanaka, 2016; Marques et al., 2019). In *P. mytiligera*, amputation initiated extensive cell division in proximity to the amputation line, resembling a blastema formation. Proliferating cell were also found in regenerating structures at later stages of regeneration, suggesting their direct involvement in the formation of the new tissue (see Figure 7 for summary). Undifferentiated circulatory cells were shown to be involved in tissue regeneration in solitary and colonial ascidians (Rinkevich et al., 2006; Auger et al., 2010; Jeffery, 2014, 2015, 2019). In colonial Styelidae species, a population of pluripotent or multipotent undifferentiated circulatory cells contributes to the formation of somatic tissues during budding and whole body regeneration (Laird et al., 2005; Voskoboynik et al., 2007; Brown and Swalla, 2012; Kassmer et al., 2020). In the solitary ascidian *C. intestinalis*, injury-induced regeneration resulted in the proliferation of circulatory cells located in the branchial basket. These cells were also labeled with alkaline phosphatase and anti-piwi antibody, indicating their undifferentiated state (Auger et al., 2010; Jeffery, 2014). In *P. mytiligera*, the branchial basket showed high level of EdU+ cells along the regeneration process. In addition, all dissected body fragments contained part of the branchial basket and the circulatory cells enclosed within it. While it is remained to be determined if these cells originated in the branchial basket or merely use it as a means of transport to the regenerating area, the high numbers of proliferating cells found in the branchial basket following amputation indicate a possible role in anterior regeneration.

While we focused on the cell division in the body wall and branchial basket in the current study, proliferating cells were also found in other structures as the digestive system and endostyle. The digestive system of the control animals as well as in animals in different stages of regeneration showed EdU positive cells. These cells, however, are probably unrelated to the regeneration signals, as intestinal and stomach tissues are continuously being replaced as part of their normal homeostasis (Ermak, 1981; Jeffery, 2014). The endostyle, which serves as a stem-cell niche in colonial species (Voskoboynik et al., 2008), showed EdU labeling at the early stages of regeneration, implying a local cell proliferation and its possible involvement in regeneration. However, it is important to note that the results of the DV axis amputation experiment indicate that the endostyle is not essential for regeneration to occur, as the dorsal body part was still able to complete the regeneration process following endostyle removal.

Among ascidians, the ability to regenerate amputated body fragments into separate individuals is strongly associated with coloniality and asexual development, as no solitary species has to date been observed to possess such robust abilities (Jeffery, 2015; Kassmer et al., 2019). The model system, *C. intestinalis*, is capable of regenerating its anterior structures, such as the siphons and neural complex from the posterior parts; whereas the anterior parts failed to regenerate posterior structures such as the heart and digestive system, and eventually decomposed (Jeffery, 2014). *P. mytiligera*’s bidirectional regeneration and unusual ability to regenerate all tissue types and organ systems distinguishes it from other solitary ascidian species studied so far (see Supplementary Table 4 for summary) and suggests the activation of regeneration programs that might be compromised or inhibited in other solitary species (Liu et al., 2013; Sikes and Newmark, 2013).

### *P. mytiligera*’s High Regeneration Abilities Might Constitute a Pre-adaptation (Exaptation) Trait for a Colonial Life-Style

*P. mytiligera*’s reproduction and developmental processes resemble those of most solitary ascidians and no indication of asexual development has been found for this species (Gordon et al., 2020). However, its ability to create “clones″ following dissection led us to further question its phylogenetic position.

The Stolidobranchia common ancestor is believed to have been solitary, and coloniality is assumed to be a derived life-style (Mukai et al., 1978; Zeng et al., 2006). The Styelidae is the only stolidobranch family composed of colonial and solitary species, with both presenting a wide range of developmental and regeneration processes (Alié et al., 2020). Phylogenetic analyses have indicated several independent acquisitions of coloniality in this group (Kott, 1985, 2005; Pérez-Portela et al., 2009; Alié et al., 2018, 2020). Further support for multiple transition events from a solitary to a colonial life-style comes from species that have diverged from the classical solitary or colonial characteristics, and which present intermediate morphological and developmental features. For example, solitary species of *Polycarpa* and *Dendrodoa* genera present colonial characteristics such as viviparity, a typical colonial feature (Millar, 1954, 1962; Svane and Young, 1989; Pérez-Portela et al., 2009).

Our phylogenetic results agree with Alié et al. (2018) and separate the *Polycarpa* genus from the exclusively solitary clade, placing it in a single mixed clade composed of colonial and solitary species. This topology suggests that the last common ancestor of the mixed clade was a solitary animal from which coloniality evolved at a later stage (Alié et al., 2018). According to this scenario, high regeneration abilities, as presented by *P. mytiligera*, might be a pre-adaptation (exaptation) trait for colonial life-style. The position of the colonial *Polyandrocarpa zorritensis* among members of the genus *Polycarpa* is especially intriguing, as it further supports the genetic similarity of *Polycarpa* to highly regenerative colonial species, as well as indicating a recent transition event from solitary to colonial form in this family (Alié et al., 2018; Scelzo et al., 2019).

To date, ascidian whole-body regeneration has been considered a colonial feature, as no solitary species had been shown before to possess such robust abilities. *P. mytiligera*’s ability to regenerate each individual body fragment into a whole animal has the potential to separate regeneration specific pathways from asexual development programs.

Our present findings present a valuable new model system for comparative developmental studies seeking to elucidate the evolution of regeneration and coloniality among the chordates.

## Data Availability Statement

The datasets generated for this study can be found in the online repositories. The names of the repository/repositories and accession number(s) can be found below: Sequence data have been deposited under: https://www.ncbi.nlm.nih.gov/bioproject/660913. The phylogenetic datasets are available at: https://github.com/dorohuchon/Polycarpa_mytiligera_transcriptome. The maximum likelihood trees, the alignments, and the transcriptome assembly have been deposited at https://github.com/dorohuchon/Polycarpa_mytiligera_transcriptome.

## Author Contributions

TG collectedand cultured the animals, performed the regeneration, and EdU experiments, as well as the light and confocal microscopy, drew the figures, carried out the statistical analysis, and prepared the histological sections. TG and LM analyzed the histological sections and microscopy images and interpreted the regeneration processes. AKU and DH performed the transcriptome and phylogenetic analyses. TG and NS conceived the study and interpreted the data. NS supervised the study and drafted the manuscript together with TG. All authors contributed to the article and approved the submitted version.

## Conflict of Interest

The authors declare that the research was conducted in the absence of any commercial or financial relationships that could be construed as a potential conflict of interest.
