# Supplementary material for: And Then There Were Three…: Extreme Regeneration Ability of the Solitary Chordate Polycarpa mytiligera
Source: Front Cell Dev Biol. 2021 Apr 15;9:652466. doi: 10.3389/fcell.2021.652466 (PMC8083962; doi:10.3389/fcell.2021.652466)
Supplement: Supplementary file 1 [file Data_Sheet_1.docx]

# Supplemental Information

**Table S1. Regeneration along the AP axis following a single amputation.** List of the remaining and regenerating organs in each body fragment at different time points along the regeneration process.

|  | Remaining structures/ organs systems  1 hpa | wound closure | Regenerating organs | |
| --- | --- | --- | --- | --- |
|  |  |  | 7 dpa | 30 dpa |
| Anterior fragment | - Oral siphon - Atrial siphon - Nervous system (neural complex) - Branchial basket and Endostyle (anterior part) - Digestive system (anterior intestine and anus) | 7 dpa | - Branchial basket and Endostyle (anterior part) | - Digestive system (esophagus, stomach, intestine and anus) - Heart |
| Posterior fragment | - Branchial basket and Endostyle (posterior part) - Digestive system (esophagus, stomach, intestine) - Heart | 7 dpa | - Branchial basket and Endostyle (posterior part) | - Oral siphon - Atrial siphon - Nervous system (neural complex) - Digestive system (anterior intestine and anus) |


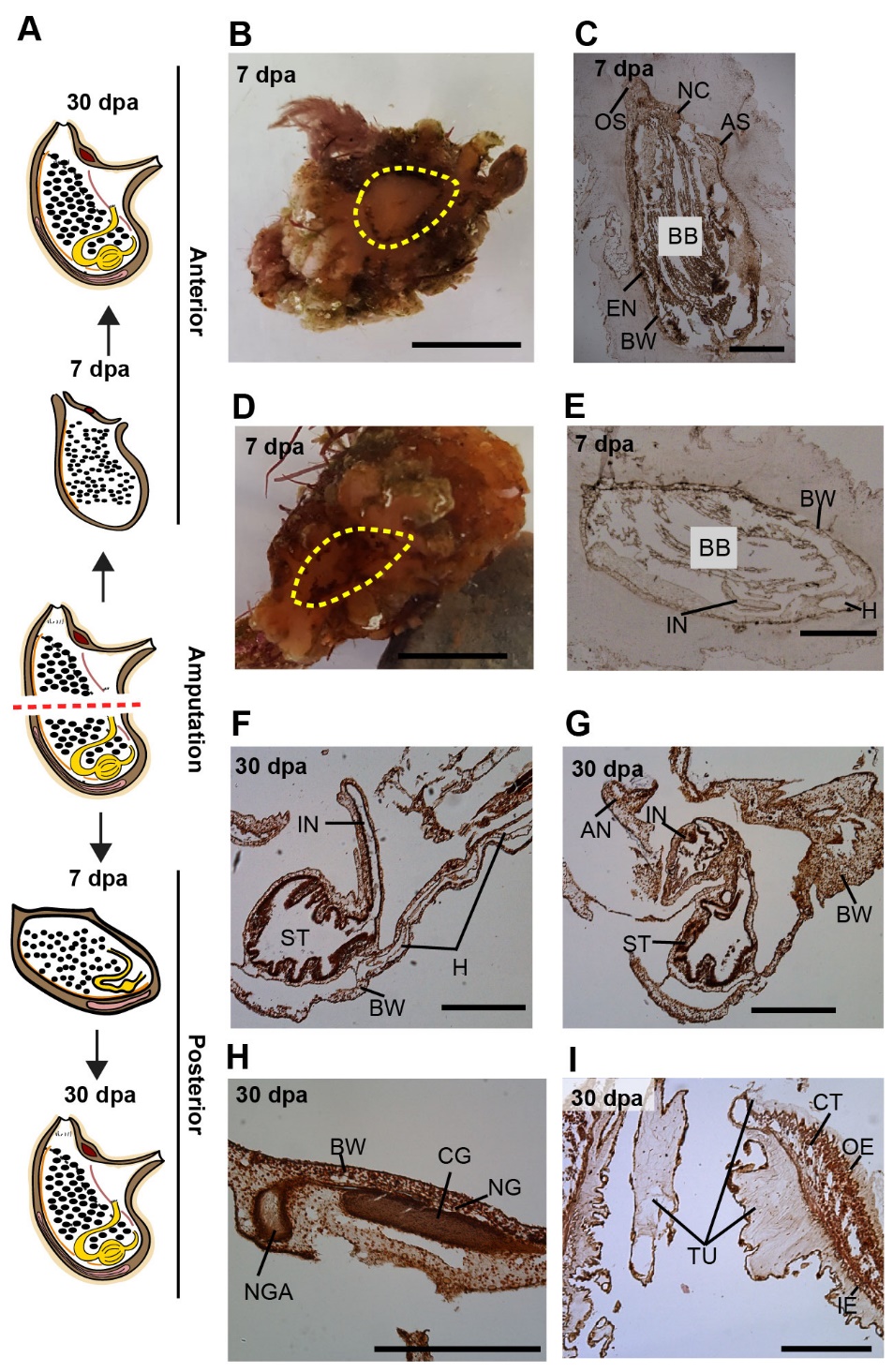


**Figure S1. Organs morphology and wound repair during regeneration along the AP axis of two body fragments.**

(**A**) Illustration depicting the regeneration process at 7 and 30 dpa. (**B-E**) wound closure at 7 dpa. (**B-C**) Anterior fragment, (**B**) *In vivo* image, Bottom view. Yellow dashed lines indicate wound border. (**C**) Histological section. (**D-E**) Posterior fragment, (**D**) *In vivo* image, top view. (**E**) Histological section. (**F-G**) Enlargements of regenerating organs at 30 dpa. (**F-G**) Anterior fragment showing the regenerated heart (H) and digestive system, including a stomach (ST), intestine (IN) and anus (AN). (**H-I**) Posterior fragment showing the regenerated neural complex in (**H**). Note the cerebral ganglion (CG), neural gland (NG) and neural gland aperture (NGA). (**I**) Regenerated oral siphon composed of connective tissue (CT), inner (IE) and outer (OE) epidermis and tunic (TU). Atrial siphon (AS), oral siphon (OS) branchial basket (BB) and endostyle (EN). Scale bar in A-C, L, N: 3 mm; in I, P-R: 500 µm; in J: 250 µm; in E, G, K: 1 mm; in Q, S: 2 mm; in S: 200 µm.

**Table S2. Regeneration along the AP axis following amputation at two levels.** List of the remaining and regenerating organs in each body fragment at different time points along the regeneration process.

|  | Remaining structures/ organs systems  1 hpa | wound closure | Regenerating structures/ organs  40 dpa |
| --- | --- | --- | --- |
| Anterior fragment | - Oral siphon - Atrial siphon - Nervous system (neural complex) - Branchial basket and Endostyle (anterior part) | 14 dpa | - Digestive system (esophagus, stomach, intestine and anus) - Heart |
| Middle fragment | - Branchial basket and Endostyle - Digestive system (anus) - Heart (anterior part) | 14 dpa | - Oral siphon - Atrial siphon - Nervous system (neural complex) - Digestive system (esophagus, stomach, intestine) |
| Posterior fragment | - Branchial basket and Endostyle (posterior part) - Digestive system (esophagus, stomach, intestine) - Heart (posterior part) | 14 dpa | - Oral siphon - Atrial siphon - Nervous system (neural complex) - Digestive system (anterior intestine and anus) |


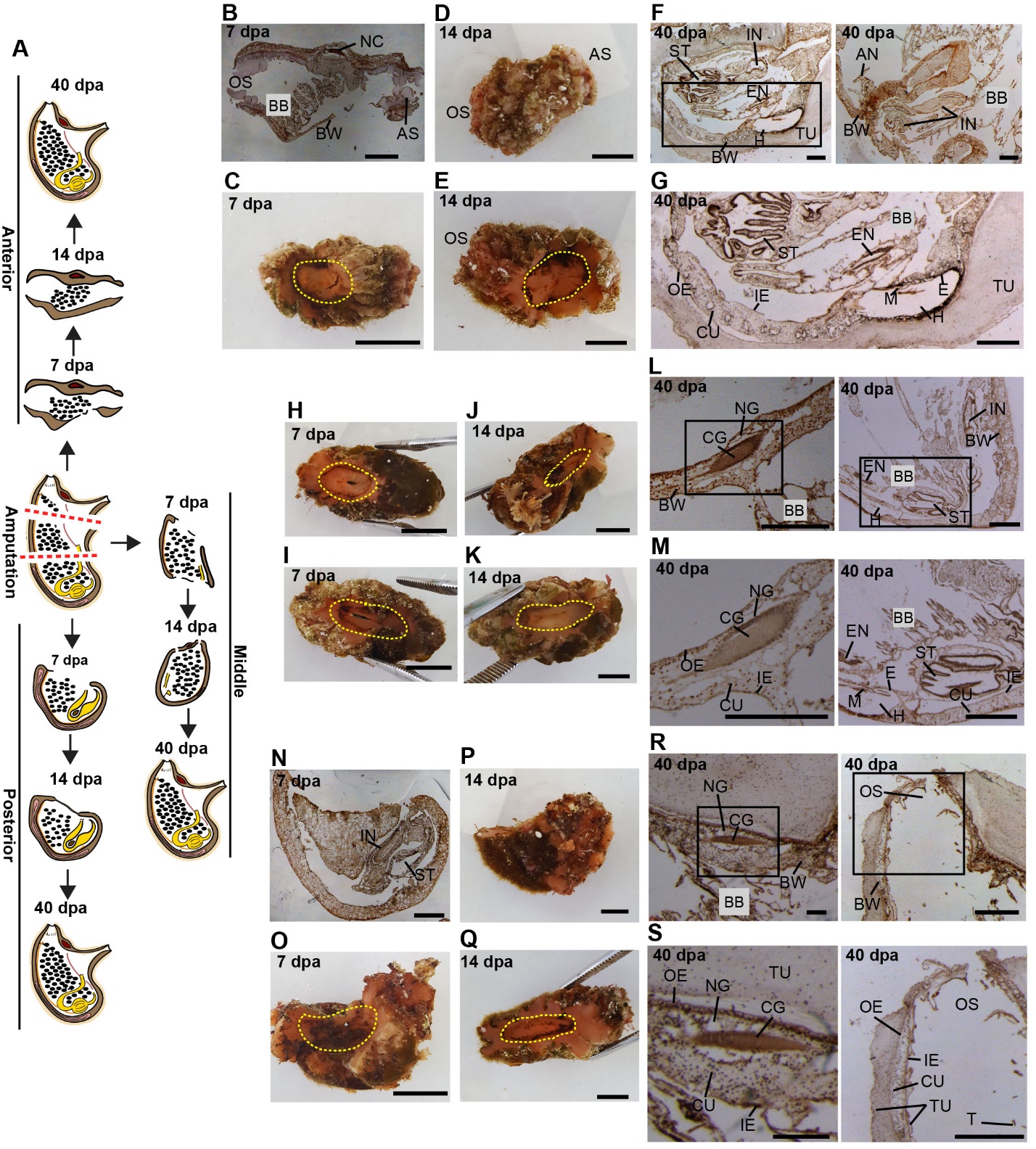


**Figure S2. Organ morphology and wound repair during regeneration along the AP axis of three body fragment.**

(**A**) Illustration depicting the regeneration process at 7, 14 and 40 dpa.

(**B-D**) Anterior body part (**B-C**) 7 dpa, (**B**) section, left view, (**C**) *in vivo* image, bottom view. Yellow dashed lines indicate wound border. (**D-E**) 14 dpa, *in vivo* image, (**D**) left and (**E**) bottom view. (**F-G**) Enlargements of regenerating organs at 40 dpa showing the digestive system and heart Note the stomach (ST), intestine (IN), and anus (AN). Enlargement of the square area shown in (**G)**.

(**H-M**) Middle body part, (**H-I**) 7 dpa *in vivo* images, top and bottom view, (**J-K**) 14 dpa *in vivo* images, top and bottom view. Note the closed wound. (**L-M**) Enlargements of regenerating organs at 40 dpa showing the regenerated neural complex composed of the cerebral ganglion (CG) and neural gland (NG). Note the regenerated heart divided to the epicardium layer (E) and myocardium (M), and the new cerebral ganglion (CG) and digestive system. Enlargement of the square areas shown in (**M)**.

(**N-S**) Posterior body part, (**N-O**) 7 dpa, (**N**) section, left view and (**O**) *in vivo* image, top view. (**P-Q**) 14 dpa *in vivo* images (**P**) left and (**Q**) top view. (**R-S**) Enlargements of regenerating organs at 40 dpa showing the regenerated cerebral ganglion and neural gland and the regenerated oral siphon composed of the connective tissue (CT), inner (IE) and outer (OE) epidermis. Enlargement of the square areas shown in (**S**).

Atrial siphon (AS), tentacles (T) and tunic (TU). Red dashed lines indicate the amputation line. Scale bar in A, C, E, G, I, K, M, O, Q: 2 mm; S-X.1: 1 mm.

|  | Remaining structures/ organs systems  1 hpa | wound closure | Regenerating structures/ organs  40 dpa |
| --- | --- | --- | --- |
| Dorsal fragment | - Oral siphon (dorsal part) - Atrial siphon - Branchial basket (dorsal part) - Digestive system (esophagus, intestine and anus) - Nervous system (neural complex) | 14 dpa | - Oral siphon (anterior part) - Digestive system (intestine and stomach) - Branchial basket (anterior part) and endostyle - Heart |
| Ventral  fragment | - Oral siphon (ventral part) - Branchial basket (anterior part) and endostyle - Heart - Digestive system (intestine and stomach) | 14 dpa | - Oral siphon (dorsal part) - Atrial siphon - Branchial basket (dorsal part) Nervous system (neural complex) - Digestive system (esophagus, intestine and anus) |

**Table S3. Regeneration along the DV axis following a single amputation.** List of the remaining and regenerating organs in each body fragment at different time points along the regeneration process.


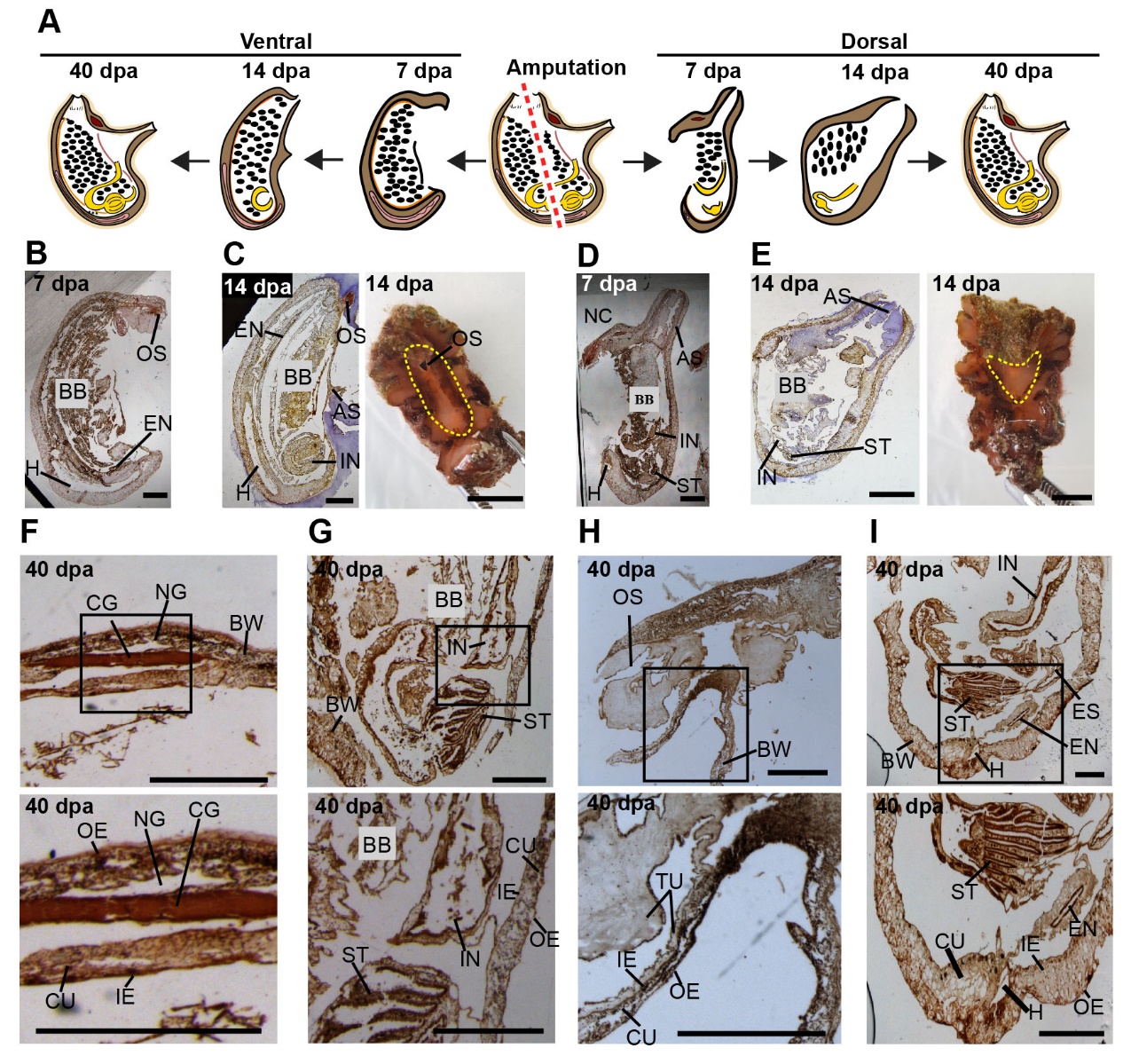


**Figure S3. Organs morphology and wound repair during regeneration along the DV axis of two body fragments.**

(**A**) Illustration depicting the regeneration process at 7, 14 and 40 dpa.

(**B-C**) Ventral body part (**B**) 7 dpa, section. Note that the wound is still open. (**C**) 14 dpa, section and *in vivo* image, posterior view. Yellow dashed lines indicate wound border. (**D-E**) Dorsal body part (**D**) 7 dpa, section. Note that the wound is still open. (**E**) 14 dpa, Section and *in vivo* image, posterior view. (**F-G**) Enlargements of ventral body fragment regenerating organs at 40 dpa showing the regenerated cerebral ganglion (CG) and neural gland (NG) in (**F**) and digestive system and heart (H) in (**G**). Note the stomach (ST), intestine (IN), and anus (AN). (**H-I**) Enlargements of dorsal body fragment regenerating organs at 40 dpa showing the regenerated oral siphon (**H**) composed of the connective tissue (CT), inner (IE), outer (OE) epidermis and tunic (TU). (**I**) Regenerated digestive system includes the stomach and heart (H).

Atrial siphon (AS), anus (AN), branchial basket (BB), endostyle (EN). Red dashed lines indicate the amputation line. Scale bar in A, C, E, G, I, K: 3 mm; L-S: 1 mm.


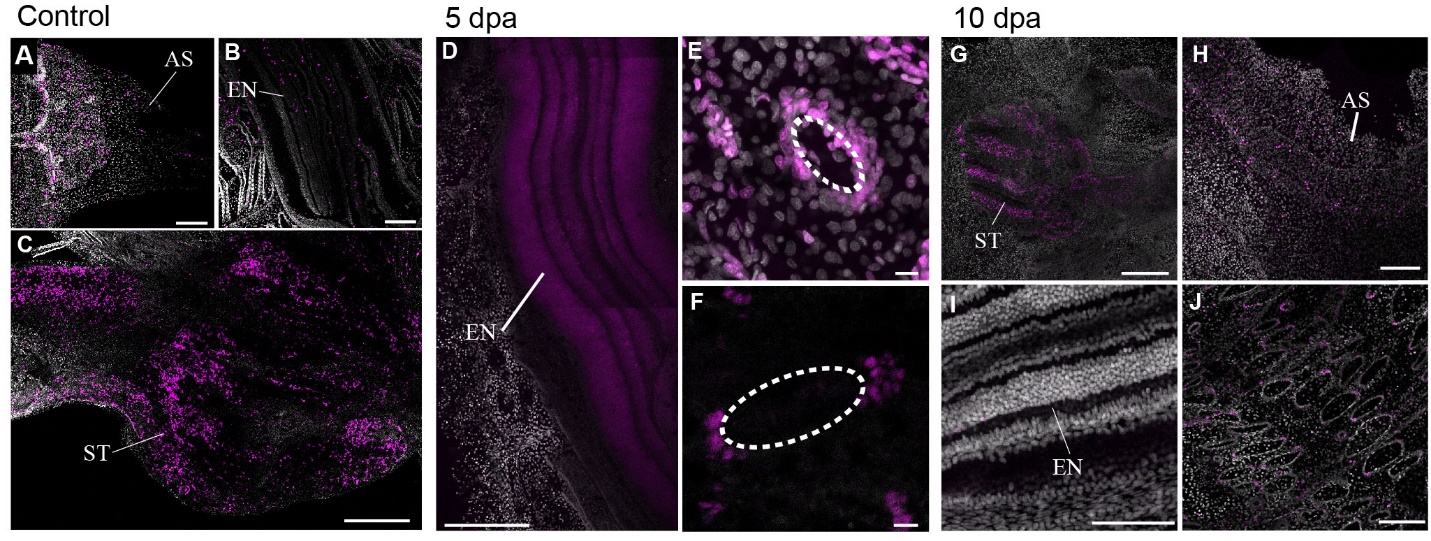


**Figure S4. Cell proliferation pattern during anterior regeneration**

Whole mount EdU staining images of anterior regeneration. EdU labeling in magenta and Hoechst nuclear staining in gray.

**(A-C)** Control. (**A**) atrial siphon, (**B**) endostyle and (**C**) stomach. **(D-F)** 5 dpa. (**D**) Endostyle, (**E**) stigma proximal to the amputation line. White dashed lines outline the stigma. (**F**) distal from the amputation line. **(G-J)** 10 dpa. (G) Stomach, (H) atrial siphon (I) endostyle (J) stigmata proximal to the amputation line.

Atrial siphon (AS), endostyle (EN), oral siphon (OS) and stigmata (ST). Scale bar: A, B, 1, H, J 100 µm; C, D, G 200; E, F 10 µm.


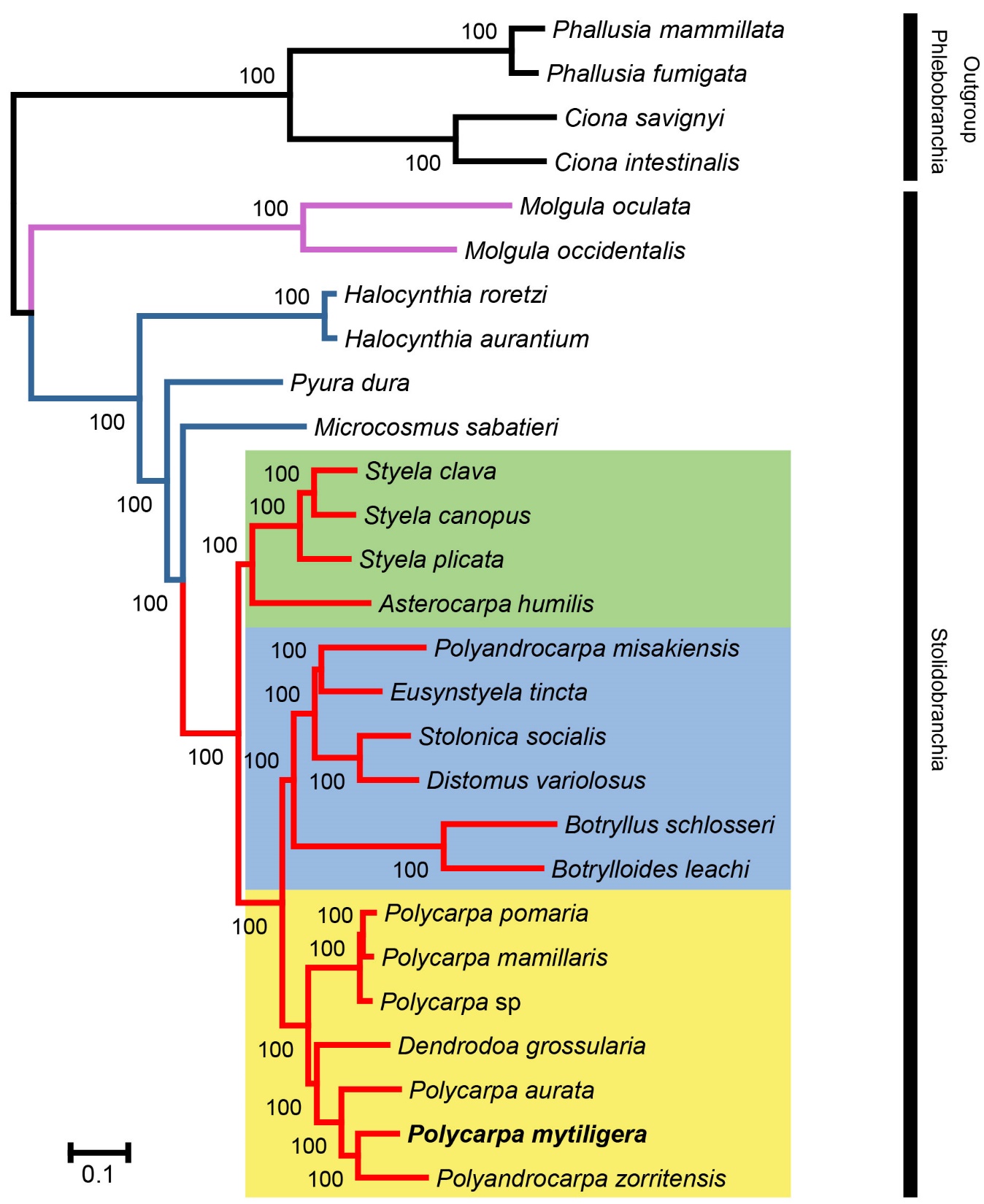


**Figure S5. Phylogenetic relationships between Stolidobranchia ascidians.** Inferred from 3341 protein coding genes under the LG+C60+F+G model. Newly sequenced *P. mytiligera* is shown in bold. Bootstrap supports are indicated near the corresponding nodes. Red branches indicate Styelidae species, blue Pyuridae species, purple Molgulidae species and black Phlebobranchia species used as outgroup. Colored squares represent different clades.

| Species | Regeneration axes | Siphons | Neural complex | Branchial basket | Digestive system | Heart | Regeneration illustration |
| --- | --- | --- | --- | --- | --- | --- | --- |
| *Polycarpa mytiligera* | Bidirectional  Anterior- posterior  Dorsal- ventral | Yes | Yes | Yes | Yes | Yes | 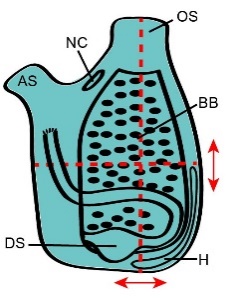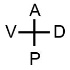 |
| *Ciona* *intestinalis/robusta* | Unidirectional  Anterior- posterior | Yes | Yes | Yes | No | No | 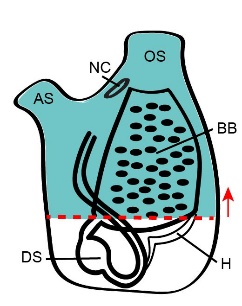 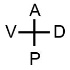 |
| *Styela plicata* | Unidirectional Anterior- posterior | Yes | Yes | N/A | N/A | N/A | 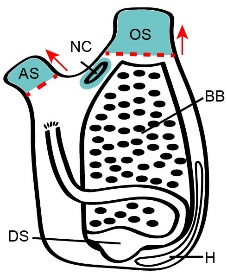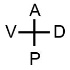 |
| *Microcosmus*  *exasperatus* | Unidirectional Anterior- posterior | Yes | No | N/A | N/A | N/A | 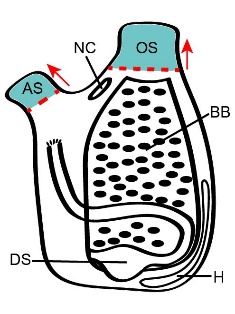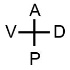 |
| *Herdmania momus* | Unidirectional Anterior- posterior | Yes | No | N/A | N/A | N/A | 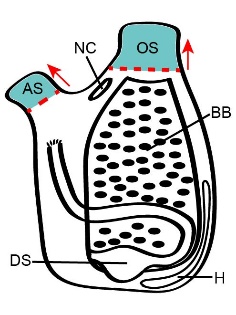 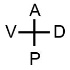 |

**Table S4. Summary of regeneration axes and regenerative body structures in studied solitary ascidians species.** List of the main studied solitary species and their regeneration abilities as documented in the literature. Regenerative body parts are highlighted in blue; N/A: not available.

*reviewed in (Jeffery, 2014, 2015, 2019; Medina et al., 2015; Gordon et al., 2019)
